# Electrospun Nanofiber Scaffolds Loaded with Metal-Based Nanoparticles for Wound Healing

**DOI:** 10.3390/polym16010024

**Published:** 2023-12-20

**Authors:** Zheng Dang, Xuemei Ma, Zihao Yang, Xiaohu Wen, Pengxiang Zhao

**Affiliations:** Faculty of Environment and Life, Beijing University of Technology, Beijing 100124, China; dangzheng@emails.bjut.edu.cn (Z.D.); xmma@bjut.edu.cn (X.M.); yzh810@163.com (Z.Y.); wxh19980813@163.com (X.W.)

**Keywords:** electrospinning, nanofiber scaffolds, extracellular matrix, metal-based nanoparticles, nanostructure, wound healing

## Abstract

Failures of wound healing have been a focus of research worldwide. With the continuous development of materials science, electrospun nanofiber scaffolds loaded with metal-based nanoparticles provide new ideas and methods for research into new tissue engineering materials due to their excellent antibacterial, anti-inflammatory, and wound healing abilities. In this review, the stages of extracellular matrix and wound healing, electrospun nanofiber scaffolds, metal-based nanoparticles, and metal-based nanoparticles supported by electrospun nanofiber scaffolds are reviewed, and their characteristics and applications are introduced. We discuss in detail the current research on wound healing of metal-based nanoparticles and electrospun nanofiber scaffolds loaded with metal-based nanoparticles, and we highlight the potential mechanisms and promising applications of these scaffolds for promoting wound healing.

## 1. Introduction

The skin is composed of three primary layers: the epidermis, the dermis, and the subcutaneous tissue. The epidermis, the outermost layer of the skin, is a keratinized stratified squamous epithelium [1]. It is relatively thin, avascular, and does not contain blood vessels [2]. The dermal layer is intimately connected to the epidermis, and predominantly consists of fibroblasts and the extracellular matrix (ECM) they produce [3]. The ECM is a dynamic three-dimensional network surrounding the cells, composed of a variety of macromolecules such as collagen, proteoglycans, and adhesion proteins [4,5]. It provides mechanical support to the cells, and plays a role in their physiological and biochemical activities [6].

Within a day after skin injury, the body’s coagulation cascade is initially activated, leading to the formation of blood clots, while platelets continuously release signaling molecules that attract inflammatory cells to the wound site [7]. During the inflammatory phase (1–2 days), neutrophils arrive at the wound to phagocytose foreign material and release cytokines that promote the proliferation and migration of monocytes and fibroblasts [8,9]. Macrophages discharge a plethora of enzymes and cytokines to clear necrotic tissue, remodel the ECM, and foster collagen and angiogenesis [10,11]. In the proliferative phase (3–7 days), fibroblasts appear around the wound, proliferate, and differentiate to produce collagen, proteoglycans, and build a new ECM, which creates a conducive environment for the regeneration of tissue and, in conjunction with signaling molecules, regulates the growth, migration, and differentiation of cells. Therefore, the formation of the ECM is crucial for wound healing, which will be elaborated on in detail later in this review [11,12]. Finally, the remodeling phase of the wound may persist for an extended period (1 week to several weeks), during which granulation tissue re-epithelializes and the ECM is continuously synthesized and remodeled, allowing the new tissue to gradually regain a state close to that of normal skin [13,14]. Wound healing is an intricate and overlapping process, and rapid and effective treatment methods for wounds are currently a significant clinical need.

Metal-based nanoparticles (MBNPs), as emerging nanobiomaterials, have good biocompatibility, and their nanoscale size enables them to be easily taken up by cells. The metal ions produced by MBNPs can participate in normal cellular activities as biologically active molecules. These properties make MBNPs widely used in drug delivery, biological imaging, and cancer treatment [15,16]. Studies have shown that during different stages of wound healing, MBNPs and their released ions exhibit unique biological properties. For example, in the early stages of wound healing, AgNPs show excellent antibacterial and anti-inflammatory properties, preventing deterioration of the wound [17]. During the inflammatory and proliferative phases, AuNPs can exert anti-inflammatory and antioxidant effects, and promote tissue regeneration [18,19]; CuNPs mainly participate in the regulation of signaling molecules related to angiogenesis and ECM reconstruction [20,21]. However, considering the potential toxic side effects of MBNPs, the application of these nanoparticles as functional components necessitates their integration with appropriate carriers to mitigate risks [22,23]. Therefore, in recent years, researchers have often combined MBNPs with electrospinning technology to prepare regenerative scaffolds. The biological functions of MBNPs are leveraged to repair wounds. The scaffolds can wrap and fix MBNPs, enabling them to be released in situ and in a controllable manner at the wound site, further enhancing the wound healing effect.

Many regenerative scaffolds for wound healing have been developed, among which the use of nanofibers to prepare tissue engineering scaffolds is a very promising solution [24]. Electrospinning is a technique for the one-step preparation of polymer nanofibers based on electrostatic forces. Nanofibers produced by this method can uniquely assist in wound healing on the basis of the following: 1. having dimensions similar to fibrin fibers, which facilitate cell adhesion and migration [25]; 2. possessing high porosity and surface area, enabling a nanoscale simulation of the fibrous network structure of the ECM, beneficial for cell proliferation and signal transduction [26,27]; 3. exhibiting good biocompatibility and mechanical strength, which do not cause significant immune rejection reactions and can support the extensive proliferation of new cells in the early stages of wound healing, promoting the formation of new tissue [28]. Additionally, by changing the spinning parameters or combining electrospinning with mixed spinning, coaxial spinning, surface modification, and other methods, scaffolds can be generated with specific structures and specific physicochemical properties [29]. When used as carriers, functional components can be encapsulated by the nanofibers through direct mixing, enabling the controlled release of these components during application [30,31]. Therefore, electrospun fibers, with their ability to mimic the ECM and serve as carriers for various functional components that promote wound healing, have become a focal point of numerous studies.

To the best of our knowledge, a review article focused on advances in the development of metal-based nanoparticles supported by electrospun nanofiber scaffolds in wound healing has not been published to date. Therefore, the aim of this review is to summary the developments about electrospun nanofiber scaffolds loaded with metal-based nanoparticles and their application in wound healing over the last five years. The present review introduces the main functions of the ECM in various stages of wound healing. Next, the key characteristics of electrospun nanofiber scaffolds are highlighted for biomedical applications. Subsequently, the study discusses the emerging nanomaterials, MBNPs, and how they can be combined with electrospinning to synergistically promote wound healing. The focus is on the existing research regarding electrospun nanofiber scaffolds loaded with MBNPs for wound healing. Finally, conclusions and future developments and challenges are presented for the field of electrospun nanofiber scaffolds loaded with MBNPs for wound treatment. An exhaustive bibliographic search was conducted using Web of Science databases. The selected studies are from 2018 and after.

## 2. ECM Dynamics during Wound Healing

**Figure 1:**
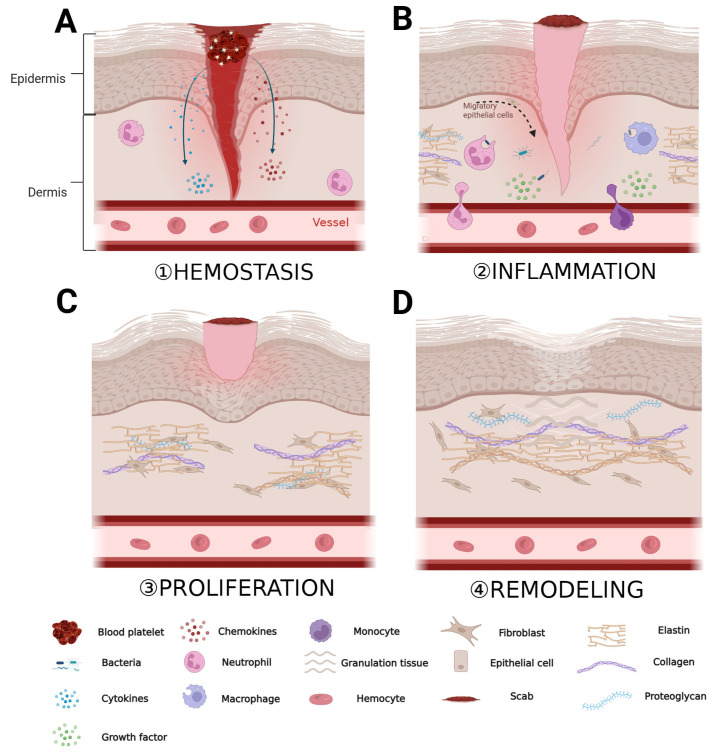
Role of ECM in the various stages of wound healing. (**A**) Period of hemostasis; (**B**) period of inflammation; (**C**) period of proliferation; and (**D**) period of remodeling. “Created with BioRender.com.” Agreement number: HW262OMTG1.

The ECM is composed of a tightly organized network of nanoscale fibers, including collagen fibers, elastic fibers, fibronectin, and other components; it plays a crucial role in various stages of wound healing [3]. The process of wound healing generally consists of four stages: hemostasis, inflammation, proliferation, and remodeling [32]. When the skin is injured, platelets aggregate around the wound and, together with fibrinogen in the ECM, form a blood clot. This clot releases a large quantity of cytokines and chemokines, attracting inflammatory cells and fibroblasts (Figure 1A) [33]. During the inflammation stage, inflammatory cells such as neutrophils, monocytes, and macrophages, as well as fibroblasts, migrate to the wound area through blood vessels and the ECM. Inflammatory cells clear pathogens and necrotic tissue at the wound site and release cytokines to promote fibroblasts to produce large quantities of ECM components, including collagen, hyaluronic acid, and proteoglycans. These ECM components provide support and protection for the damaged tissue (Figure 1B) [34,35]. In the proliferation phase, the newly formed ECM provides mechanical support and a survival environment for cells. Cells proliferate and differentiate extensively around the wound, filling the gap in the wound (Figure 1C) [36]. Finally, granulation tissue forms around the wound and new blood vessels are formed. Wound healing enters the remodeling phase, during which the granulation tissue undergoes epithelialization, and the ECM continuously remodels, gradually restoring the newly formed tissue to a state closer to normal tissue (Figure 1D) [3,37].

**Figure 2:**
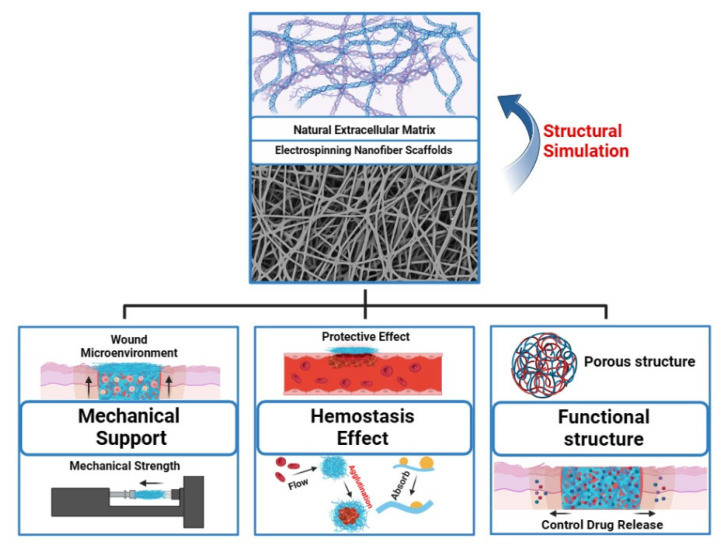
Multifunctional electrospun nanofiber scaffolds for wound healing applications. “Created with BioRender.com.” Agreement number: TI2684VL3Q.

The ECM plays a crucial role in the normal healing process of wounds. Electrospun nanofiber scaffolds mimic the intricate structure of the skin’s ECM at the microscale and provide robust mechanical support to promote cell proliferation, adhesion, and migration at the wound site. Additionally, the high surface area-to-volume ratio of nanofibers can aid in rapid hemostasis of the wound [38]. Incorporating functional materials into nanofibers, such as antimicrobial agents, growth factors, and metallic materials, provides additional biological functionalities that help further promote wound healing (Figure 2).

## 3. Electrospun Nanofiber Scaffolds in Tissue Engineering

Electrospinning is a technique that uses a polymer solution to prepare nanofibers [39]. In general, the basic equipment for electrospinning mainly includes a high-voltage power supply, a spinneret, an injection pump, and a collector plate [40]. During operation of the device, the injection pump pushes the polymer solution in the syringe to the spinneret connected to the high-voltage power supply. Under the action of surface tension, the polymer solution forms charged droplets at the nozzle of the high-voltage power supply. The electrostatic repulsion generated by the surface charge on the droplet surface is opposite to the direction of the surface tension. As the voltage intensity gradually increases, the polymer droplets are elongated under the action of the electrostatic repulsion produced by the surface charge, forming a Taylor cone [29,41]. When the electrostatic repulsion on the surface of the polymer droplet exceeds the surface tension, the droplet is elongated in the electric field, forming a charged jet. Finally, the solvent in the polymer solution evaporates, forming polymer nanofibers. The nanofibers accumulate on the collector plate in a random direction, forming a nanofiber scaffold [42,43].

**Figure 3:**
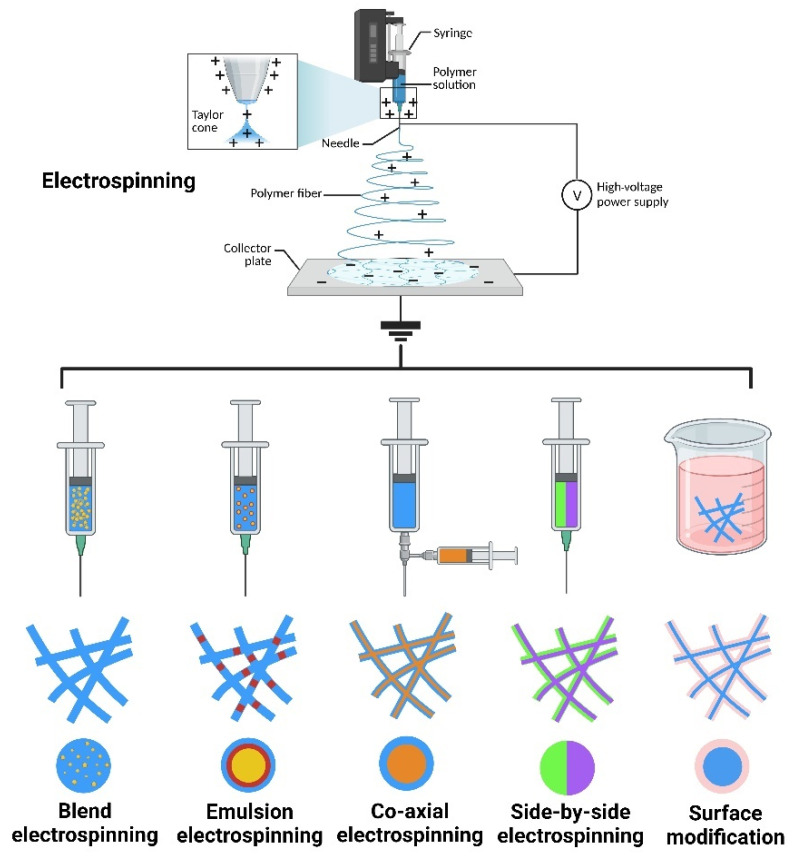
The method and production of nanofiber. “Created with BioRender.com.” Agreement number: LZ2684T4E0.

Additionally, different structures and functions of scaffolds can be obtained by adjusting the spinning solution, spinning parameters, spinning environment, and other conditions [44]. When functional materials are incorporated, polymer nanofiber scaffolds can be prepared by various electrospinning techniques based on the type of functional material and solvent requirements, such as blend electrospinning, coaxial electrospinning, emulsion electrospinning, and side-by-side electrospinning, along with surface modifications, producing special structures such as the core-shell, multilayer, and Janus structures. This enables the controlled release of functional materials, such as burst release, sustained release, and multiple release, depending on the specific application (Figure 3) [45,46]. Electrospun nanofiber scaffolds possess high interconnectivity and a high specific surface area, and their porous three-dimensional structure exhibits excellent mechanical strength and contains multiple cell binding sites, which can promote the interaction between cells and the ECM when applied at an injury site [47]. During tissue remodeling, degradable nanofiber scaffolds are gradually decomposed and absorbed, without adverse effects on the structure and function of the tissue. Based on the above description, electrospun nanofiber scaffolds meet the ideal conditions for constructing tissue engineering scaffolds, as summarized in Table 1, and are ideal candidates for preparing artificial skin, wound dressings, and skin tissue scaffolds. Currently, increasingly more research is focused on using metal-based nanoparticles and electrospinning technology to prepare composite scaffolds for treating drug-resistant bacteria and difficult-to-heal wounds. The inherent biological functions of metal-based nanoparticles can endow scaffolds with unique biological activity and material properties. This type of bionanomaterial provides new ideas and unique solutions for preventing bacterial infections and treating difficult-to-heal wounds, and is expected to become an important therapeutic method and material selection in the biomedical field in the future [48].

## 4. Metal-Based Nanoparticles in Wound Healing

MBNPs refer to small metal particles with at least one dimension in three-dimensional space in the range of 1–100 nm [58]. MBNPs are characterized by their small size, large specific surface area, and quantum size effects. These properties result in completely different physical and chemical characteristics from macroscopic metals [59]. In the past decade, many metal-based nanoparticles (such as AgNPs, AgNPs, and CuNPs) have been extensively studied and were shown to have the advantages of simple preparation, strong stability, good biocompatibility, and good biodegradation, which are very suitable characteristics for biomedical applications [60].

**Figure 4:**
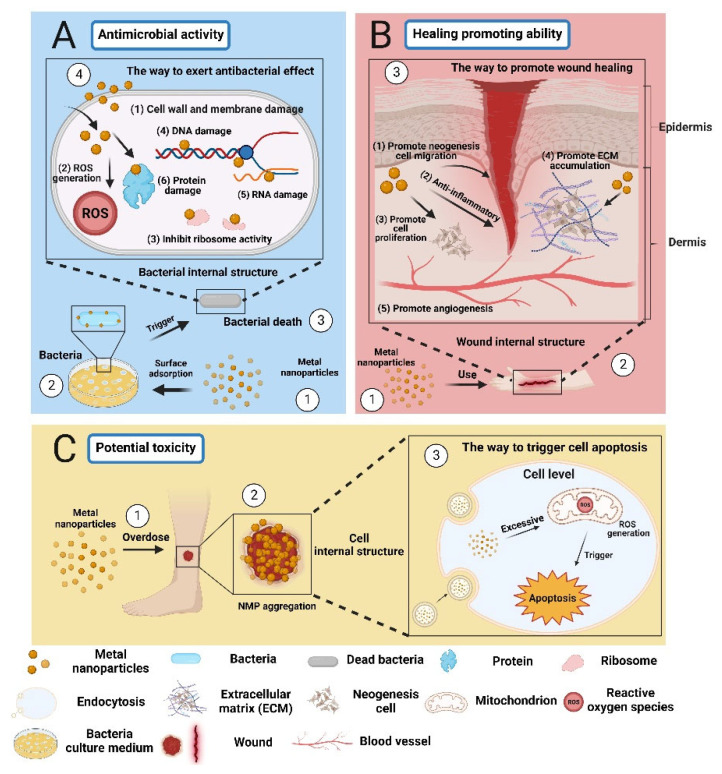
The effects of metal-based nanoparticles: beneficial and harmful aspects in wound healing. (**A**) The antimicrobial activity of MBNPs (**B**) The healing promoting ability of MBNPs (**C**) The potential toxicity of MBNPs. “Created with BioRender.com.” Agreement number: CN2684TD8X.

MBNPs can inhibit or kill microorganisms through various mechanisms. Their antibacterial mechanism differs from conventional antibiotics, which makes it difficult for bacteria to develop resistance [48,61] (Table 2). For example, 1. MBNPs with a high surface area can effectively bind to the cell wall and membrane of microorganisms, interact with the proteins on the surface, and disrupt their cellular structure [62]. 2. MBNPs can kill microorganisms by directly contacting them, inducing the production of reactive oxygen species (ROS) that can damage DNA, RNA, proteins, and other substances inside the cell (Figure 4A) [63,64]. 3. Once inside the cell, MBNPs are free to interact with cellular structures (e.g., membranes, ribosomes, proteins, DNA, RNA), disrupting cell functions [65,66]. Moreover, some metal-based nanoparticles can directly promote angiogenesis, ECM accumulation, or wound re-epithelialization through their own functions (such as anti-inflammatory, antioxidant, cell proliferation-promoting, and cytokine-regulating effects), or through combining MBNPs with photothermal therapy (PTT) and photodynamic therapy (PDT). This can improve wound healing (Figure 4B) [15,67].

However, the safety and efficacy of metal nanoparticles as functional components in medical products need to be evaluated by regulatory agencies due to their different physicochemical properties and biological effects [68]. For instance, the US FDA guidance document “Considering Whether an FDA-Regulated Product Involves the Application of Nanotechnology” emphasizes the deliberate manipulation and control of dimensions to produce specific properties. The emergence of new properties or phenomena may raise questions about safety, effectiveness, performance, quality, or public health impact, warranting further evaluation [69]. Moreover, there have been studies showing that direct application of MBNPs to the wound surface can lead to the aggregation of NPs around the wound, and high local concentrations can potentially cause toxicity and affect cell regeneration (Figure 4C) [23,62]. Therefore, in recent years, researchers have combined MBNPs with electrospinning technology, using polymer nanofibers to wrap MBNPs, which can not only use metal-based nanoparticles for in situ wound treatment but also control the release rate of nanoparticles, which has great potential in biomedical applications [70,71,72] (Table 3). In this review, research on the use of electrospun nanofibers loaded with metal nanoparticles for wound healing is clarified. The following is a brief summary of the characteristics and applications of various MBNPs.

**Table 2:** Comparative analysis of metal-based nanoparticles’ antibacterial effects.

MBNPs Type	Nanomaterial Properties	Bacterial Species	Exposure Time	Biological Activity	References
AgNPs	Spherical; 40 nm	*E. coli* MTCC 062	18 h	MIC = 3.6 µg/mL	[73]
		*P. aeruginosa* MTCC 424		MIC = 2.7 µg/mL	
	Spherical; 18.936 ± 7.789 nm	*E. coli* (ATCC25922)	24 h	MIC = 50 μg/mL	[74]
		*P. aeruginosa* (ATCC27853)		MIC = 6.25 μg/mL	
AuNPs	Spherical; 40 nm	*E. coli* (ATCC No. 25922)	24 h	MIC = 3.9 μg/mL	[75]
		*P. aeruginosa* (PTCC No. 1707)		MIC = 1.95 μg/mL	
		*S. aureus* (ATCC No. 25923)		MIC = 3.9 μg/mL	
		*B. subtilis* (ATCC No. 21332)		MIC = 15.62 μg/mL	
	Spherical; 3.5 nm	*P. aeruginosa*	24 h	MIC = 100 μg/mL	[76]
		*S. aureus*		MIC = 100 μg/mL	
		*E. coli*		MIC = 100 μg/mL	
	Star; 26.0 ± 2.6 nm	*S. aureus* (ATCC 12600)	24 h	MIC = 250 μg/mL	[77]
CuNPs	Spherical; 38 nm	*E. coli*	24 h	MIC = 350 μg/mL	[78]
		*S. aureus*		MIC = 150 μg/mL	
		*C. albicans*		MIC = 300 μg/mL	
	Spherical; 17.85 nm	*P. aeruginosa*	24 h	Z = 16.00 ± 1.63 mm	[79]
		*S. aureus*		Z = 9.67 ± 0.47 mm	
	Spherical; 11–33 nm	*S. aureus*	24 h	MIC = 31.25 μg/mL	[80]
		*B. cereus*		MIC = 62.5 μg/mL	
		*E. coli*		MIC = 125 μg/mL	
		*K. pneumoniae*		MIC = 125 μg/mL	

MIC, minimal inhibitory concentration; Z, zone of inhibition.

### 4.1. Silver-Based Nanoparticles in Wound Healing

Silver-based materials have been used since ancient times to control wound infections. With the development of nanomedicine, silver nanoparticles (AgNPs) have attracted considerable research attention due to their simple and diverse synthesis methods, nontraditional and effective antibacterial mechanisms, and low toxicity [81]. Currently, the antibacterial mechanism of silver nanoparticles is summarized as follows: 1. AgNPs absorb on and penetrate the cell wall and membrane of bacteria to destroy their structure [82]; 2. AgNPs destroy intracellular structures within bacteria, and release Ag+ into the cytoplasm that specifically binds to proteins, resulting in enzyme inactivation [83]; 3. AgNPs generate a large amount of ROS within the bacteria, leading to oxidative stress in the bacteria [84]; and 4. AgNPs induce structural and permeability changes in bacteria, leading to the dissipation of proton power and the destruction of cell membranes [85,86].

Additionally, during the inflammatory phase, AgNPs can reduce the inflammatory response and exert anti-inflammatory effects by diminishing the production of pro-inflammatory cytokines such as interleukin-8 (IL-8), interleukin-6 (IL-6), and tumor necrosis factor-alpha (TNF-α) [87,88,89,90]. Concurrently, AgNPs also promote cellular proliferation at the wound site and induce the differentiation of fibroblasts into myofibroblasts, accelerating wound closure [91,92]. Furthermore, during the remodeling phase of the wound, AgNPs facilitate the accumulation of the ECM and stimulate angiogenesis by modulating the release of signaling molecules such as transforming growth factor-beta (TGF-β) and vascular endothelial growth factor (VEGF), thereby hastening the restoration of skin structure [93,94,95] (Table 4).

Regarding the toxic effects of AgNPs, extensive research indicates that exposure to environments with high concentrations or particle sizes smaller than 10 nm can cause varying degrees of damage to most human cell lines (such as macrophages, erythrocytes, hepatocytes, etc.) [103,104,105,106,107]. These nanoparticles may also be transported via the bloodstream to organs such as the liver, spleen, kidneys, and lungs, inducing inflammation, damage, and even death in animals [108,109,110,111,112,113]. Therefore, the use of AgNPs in wound treatment necessitates finding reasonable methods to deliver AgNPs that ensure their efficacy while minimizing toxicity as much as possible.

### 4.2. Gold-Based Nanoparticles in Wound Healing

Since the 20th century, gold salt drugs have been used to treat diseases such as arthritis [114]. In the 21st century, AuNPs have become widely used in biomedical imaging, targeted therapy, antibacterial treatment, and cancer treatment due to their superior photoelectric and physical properties and biocompatibility [115,116,117]. Regarding the antibacterial activity and wound healing ability of AuNPs, Zhan et al. summarized that AuNPs have broad-spectrum antibacterial properties, and can be combined with photothermal effects to perform targeted ablation and sterilization at the affected area [118]. Furthermore, multiple studies have indicated that the large surface area and electron-accepting tendency of AuNPs enable them to interact with ROS and participate in the regulation of cytokines (such as the IL family, TNF-α) and growth factors (such as VEGF, FGF, TGF-β), exerting antioxidant and anti-inflammatory effects [119,120,121]. Additionally, they can synergistically promote wound healing through the combination of photothermal therapy (PTT) and photobiomodulation therapy (PBMT) (Table 5).

Currently, there is still much controversy surrounding the toxic effects of AuNPs. The main findings suggest that the toxicity of AuNPs is related to their size, shape, and dose (Woźniak et al. [128]; Isoda et al. [129]). However, there is a significant variation in the doses used in the different studies, and even contradictory results have been reported in different toxicity studies (Tao et al. [130]; Rambanapasi et al. [131]; Lopez-Chaves et al., [132]). Therefore, a systematic investigation is still needed to fully understand the toxic effects of AuNPs.

### 4.3. Copper-Based Nanoparticles in Wound Healing

Copper, a chemical element with atomic number 29 and symbol Cu, is a bioactive metal that has been extensively studied. In biological systems, copper is often present in the form of Cu^2+^, and plays a crucial role in the formation of various connective tissues in bones, blood vessels, as well as in lipid metabolism, carbohydrate metabolism, iron metabolism, and antioxidant defense mechanisms [133,134,135]. Despite its prominent physiological roles, an excess of copper ions in the body may lead to cellular oxidative stress, resulting in DNA and protein damage. Therefore, careful management of copper intake is necessary [136,137].

In recent years, copper-based nanoparticles have been shown to exist in the body not as ions, significantly reducing their toxicity compared to conventional copper-based materials [138,139,140,141]. Moreover, several studies have indicated that copper-based nanoparticles are more inclined to bind to bacterial cell membranes, disrupting their external structure and inducing the generation of ROS, further inhibiting bacterial growth [142,143,144]. In terms of wound healing, CuNPs can participate in the regulation of various stages of behavior, including hemostasis and the inflammatory phase, by regulating the release of platelet-derived growth factor (PDGF) and hypoxia-inducible factor-1-alpha (HIF-1), and catalyzing superoxide dismutase (SOD) activity [145,146,147,148]. During the proliferation and remodeling phase, they stimulate the production of signaling molecules such as VEGF, FGF, and TGF-β, accelerating ECM deposition and angiogenesis to promote wound healing [149,150,151,152] (Table 6).

## 5. Electrospun Nanofiber Scaffolds Loaded with Metal-Based Nanoparticles for Skin Regeneration

Currently, various metals (such as silver, gold, and copper) have been widely used in the study of MBNPs. Silver nanoparticles are the most widely used metal-based nanoparticles, and have been widely used in commercialization and wound research due to their superior antibacterial properties and green preparation methods [89]. Gold has been used in cosmetic and anti-inflammatory applications since ancient times, and because of its superior ductility and ability to combine with photothermal and targeted therapies, gold nanoparticles are often used in medical research [115]. Copper is an essential trace element in all human tissues, and is vital in multiple metabolic pathways [138]. The following section summarizes the recent research on electrospun nanofibers loaded with MBNPs for wound healing, classified by metal type.

### 5.1. Electrospun Nanofiber Scaffolds Loaded with Silver-Based Nanoparticles

Allizond et al. reported a one-step method for preparing electrospun nanofibers containing AgNPs from polylactic acid/polyethylene oxide (PLA/PEO). The experimental results showed that AgNP–PLA/PEO nanofibers effectively killed *S. epidermidis* and *E. coli* by rapidly releasing silver ions [156]. Spagnol et al. modified cellulose whiskers (CWs) to produce carboxylated CWs (CWSAc) with carboxyl groups (-COO-), and then immersed them in a silver nitrate solution (AgNO_3_) to synthesize AgNPs in situ and anchor AgNPs on the surface. They prepared CWSAc/AgNPs and combined them with electrospinning to prepare polyvinyl alcohol (PVA) antibacterial fibers loaded with CWSAc/AgNPs. The antibacterial fibers exhibited inhibition zones up to 11 mm in diameter against *E. coli*, *P. aeruginosa*, and *S. aureus* in antibacterial tests [157]. However, recent studies have found that using AgNPs alone as antibacterial agents had weaker antibacterial effects against Gram-positive bacteria. Therefore, researchers wrapped AgNPs and ciprofloxacin separately with polyvinylpyrrolidone and ethyl cellulose, respectively, and prepared Janus-structured nanofibers using side-by-side electrospinning technology. The experimental results showed that ciprofloxacin and AgNPs were wrapped on both sides of the nanofiber and were released separately in a controlled manner. Antibacterial tests showed that the synergistic effect of AgNPs and ciprofloxacin had strong antibacterial activity against Gram-positive and Gram-negative bacteria, providing a new idea for the research of novel electrospun nanofiber membranes [158].

To minimize the potential toxicity of AgNPs, researchers have recently found that plant extracts and natural polymers can be used for the in situ synthesis of AgNPs in electrospinning solutions. Kohsari et al. used sorghum extract to reduce a silver nitrate solution (AgNO_3_) to prepare “green” AgNPs, and combined them with chitosan/polyethylene oxide nanofibers through an electrospinning technique. The experimental results showed that AgNPs, when combined with electrospinning, exhibited sustained release and superior antibacterial properties, indicating their potential for practical applications [159]. El-Aassar et al. prepared biologically active AgNP spheres (Ag–PGA/HA) using polygalacturonic acid (PGA) and hyaluronic acid (HA), and loaded them onto PVA nanofibers. The antibacterial results showed that Ag–PGA/HA–PVA nanofibers exhibited significant antibacterial effects. On the eighth day of use in rat wounds, Ag–PGA/HA–PVA nanofibers significantly promoted wound healing. Subsequent histopathological results showed that collagen deposition at the wound site was tightly packed and the wound was completely epithelialized [160]. Therefore, electrospun nanofibers loaded with green AgNPs also exhibited positive effects on wound repair.

### 5.2. Electrospun Nanofiber Scaffolds Loaded with Gold-Based Nanoparticles

Gold nanoparticles have good surface modifiability and are excellent antibiotic carriers. Yang et al. developed a polycaprolactone (PCL)/gelatin nanofiber loaded with gold nanoparticles (Au–APA) modified with antibiotic drug intermediates (APA). The nanofiber had good biocompatibility, and an analysis of its healing effects on rat wound infection and antibiotic-resistant bacterial infection models showed better therapeutic effects on infected wounds than did the controls [73]. Based on the photothermal effect of gold nanoparticles, Tian et al. designed a composite nanoparticle (Au@CD) composed of AuNPs and carbon dots (N,S-CDs), which was combined with electrospinning and used for PTT in animal models. The results showed that Au@CD nanofibers had good antibacterial activity and effectively promoted collagen accumulation and blood vessel generation at the wound site, providing a new approach to promoting wound healing [161]. By leveraging the similar crystal structures of AgNPs and AuNPs, a characteristic that also makes them easily co-prepared, Bai et al. developed a sea urchin-like Au–Ag bimetallic nanoparticle-modified polyacrylonitrile (PAN) nanofiber mat. Experimental results showed that the nanofiber mat enhanced osteoblast activity, had good antibacterial activity, was nonirritating to the skin, and had a significant promoting effect on the healing of infected wounds [70]. Considering the potential toxicity of AuNPs prepared by reducing agents such as NaBH_4_, researchers have recently turned to natural polymers such as chitosan and xanthan gum to synthesize biogenic AuNPs. Then, these biogenic AuNPs were combined with drugs such as moxifloxacin hydrochloride, peppermint oil, and a nanoemulsion (SNE) to produce antibacterial and anti-inflammatory nanofibers. This approach provides a new strategy and method for the development of efficient and environmentally friendly wound healing materials [162,163].

### 5.3. Electrospun Nanofiber Scaffolds Loaded with Copper-Based Nanoparticles

Jahangirian et al. prepared PVA electrospun membranes loaded with CuNPs by blending CuNPs with PVA and CS. These membranes were used as the inner layer and combined with an outer layer of PVP electrospun membranes to form wound dressings for a full-thickness skin defect model in rats. The experimental results showed that CuNPs could be uniformly dispersed in the polymer fibers and exhibited excellent antibacterial effects against *Bacillus cereus*, *S. aureus*, *E. coli*, and *P. aeruginosa*. Furthermore, the use of PVA/CS/CuNPs composites in treating rat wounds led to rapid healing within a short period of time [164]. The “green” synthesis of CuNPs as implantable materials has been a focus of research. Fahimirad et al. designed electrospun membranes with PCL as the inner support layer and PVA as the outer covering layer. They incorporated CuNPs synthesized using Quercus infectoria galls (QLG) extract into the PVA spinning solution, resulting in PCL/PVA nanofibers loaded with QLG and CuNPs (PCL/PVA/QLG/CuNPs). Analysis using FTIR spectroscopy, XRD, and other techniques confirmed the successful synthesis of CuNPs combined with the functional components of QLG, which were uniformly dispersed in the PVA nanofibers. Cell viability, antimicrobial, and animal experiment results demonstrated that the PCL/PVA/QLG/CuNPs nanofiber membrane was completely non-toxic and exhibited significant antimicrobial activity against *S. aureus.* The wound healing effects on non-infected and infected *S. aureus* wounds were improved by 77.6% and 73.8%, respectively [165].

When used as an antimicrobial material, it is important to avoid potential toxic side effects of CuNPs by achieving controlled release during application. J. Ahire et al. incorporated CuNPs into a solution of PDLLA and PEO at a concentration of 150 mg/mL^−1^ to prepare CuNP-loaded nanofibers using a one-step method. They evaluated the release capacity of CuNPs in the nanofibers, their cytotoxicity, and antimicrobial activity against *P. aeruginosa* and *S. aureus*. The experimental results showed that CuNPs in the CuNPs/PDLLA/PEO nanofibers existed in an irregular form, and the release rate could be adjusted by varying the ratio of PDLLA and PEO. The released CuNPs exhibited significant inhibitory effects against *P. aeruginosa* and *S. aureus.* Additionally, cytotoxicity experiments demonstrated that CuNPs had toxic side effects on a small number of cell lines [166]. Therefore, achieving controlled release of NPs remains an important topic for further research and application.

## 6. Conclusions and Future Directions

Since trauma is a major health problem that all countries across the globe face, current research on new functional materials with properties that promote wound healing is making new progress every day. Based on current research, MBNPs involving gold, silver, and copper have functions that promote wound healing, such as exerting antibacterial and anti-inflammatory activity, and promoting cell proliferation, angiogenesis, and tissue remodeling [121,167,168]. Additionally, some MBNPs have expanded their biological applications through their unique properties. For example, gold nanoparticles have photothermal effects and can be used in photodynamic therapy [169,170]. Although there is considerable research on MBNPs in biomedical applications, we should also pay attention to the nanotoxicity of nanomaterials. Nanotoxicity is related to factors such as the size, shape, surface area, and structure of nanomaterials. Studies have revealed that metal nanoparticles may have certain toxicity to cells after entering the human body, and nanoparticles easily accumulate in organs such as the liver and kidneys through the bloodstream [171,172,173,174]. Therefore, before being used in clinical research in the future, MBNPs must be stabilized in a carrier through embedding, solidification, and other methods to reduce their potential toxicity [175]. Additionally, there has been controversy regarding the synthesis of MBNPs for biomedical applications in recent years, and green synthesis is considered to be a safer and friendlier synthesis for human health than traditional physical and chemical synthesis, which deserves further research [176].

Electrospinning technology can rapidly prepare large quantities of polymer nanofiber scaffolds, which can be used to cover or fill wounds for in situ treatment [177]. For example, biomimetic nanofiber scaffolds can be prepared according to the structure and materials of damaged tissue, which can provide a survival environment for new cells in the early stage of wound healing, promoting cell proliferation and migration [178]. In the future, research on nanofiber scaffolds for wound healing can focus more on the dynamic changes in the ECM in different healing stages and strive to simulate the structure of the ECM in a more detailed way. Furthermore, adding functional materials such as growth factors and antibacterial materials to nanofibers, or preparing composite nanofiber scaffolds can help regulate cell signaling pathways and achieve intelligent treatment of wounds in different healing stages.

In summary, electrospun nanofiber scaffolds loaded with metal-based nanoparticles, as a stable carrier for MBNPs, have the superior properties of nanofibers and can better treat wounds by combining the properties of MBNPs. Moreover, for the needs of different healing stages, composite and multi-level nanofibers could be prepared with different MBNPs to achieve diverse and efficient trauma treatment. These research results could provide new ideas and methods for trauma treatment and make valuable contributions to the development of nanomedicine.

## Figures and Tables

**Table 1:** Design requirements for tissue engineering scaffolds.

Design Characteristics for Scaffolds	Elaboration	References
Biocompatibility and non-toxicity	The scaffolds and the materials used should have good compatibility and not cause any adverse reactions to cells and tissues.	[49,50]
Biodegradability	The scaffolds should be gradually absorbed and de-graded by the cells and tissues in the body during the wound healing process to avoid any unnecessary side effects.	[51]
Porous structure	Scaffolds with appropriate pore sizes can provide a suitable proliferative environment, enhance cell-matrix interactions, and facilitate rapid transport of nutrients and metabolic waste within the scaffold.	[52,53,54]
Mechanical properties	The scaffold should possess mechanical properties that mimic those of native tissues, while also provid-ing a conducive microenvironment for the growth and migration of new tissues.	[55]
Surface properties	Consideration of scaffold surface hydrophilicity and morphology is crucial for optimizing cell adhesion and proliferation in tissue engineering scaffold design and fabrication.	[56,57]

**Table 3:** Compilation of patents on electrospun nanofibers loaded with metal-based nanoparticles.

Patent No.	Title	Applicant	Country Code
WO2017218692	Method of manufacturing silver nanoparticles, cellulosic fibers, and nanofibers containing silver nanoparticles, fibers, and nanofibers containing silver nanoparticles; use of silver nanoparticles for the manufacture of cellulosic fibers and nanofibers, and wound dressing containing silver nanoparticles	California Institute of Technology	US
WO2008100163	Method of manufacturing silver nanoparticles, cellulosic fibers, and nanofibers containing silver nanoparticles, and uses thereof in bactericidal yarns and tissues	Instytut Wlókien Naturalnych	PL
EP2126146	Alginate hydrogel containing nanofibers onto which antibacterial metal nanoparticles are adsorbed, used thereof, and method for manufacturing same	Inst Of Natural Fibres and Medicinal Plants	EP
KR1020200018140	Composite, antibacterial agent, and method of producing composite	The Industrial-Academic Cooperation Group of Kangwon National University	KR
JP2022184334	Nanofiber-based dental mask and manufacturing method thereof	Sugino Machine Ltd.	JP
KR1020220005303	Method of manufacturing silver nanoparticles, cellulosic fibers, and nanofibers containing silver nanoparticles, fibers, and nanofibers containing silver nanoparticles; use of silver nanoparticles for the manufacture of cellulosic fibers and nanofibers, and wound dressing containing silver nanoparticles	Woosuk University Industry-Academic Cooperation Group	KR

US: United States; EP: European; JP: Japan, PL: Poland; KR: Korea.

**Table 4:** Applications of silver-based nanoparticles in wound healing.

Operation Method	Nanomaterial Properties	Biological Activity	References
AgNPs	99 nm	Antioxidant Anti-inflammatory Cell proliferation and migration	[96]
AgNPs ointment	Spherical; 10–35 nm	Angiogenesis Antioxidant activity by suppression of ROS generation Anti-inflammatory effects via reduction of pro-inflammatory cytokine levels Promotion of ECM synthesis	[97]
AgNPs hydrogel	Spherical; 20 nm	Cell proliferation and migration Anti-inflammatory Collagen secretion	[98]
AgNPs hydrogel	Spherical; 7.2–16.8 nm	Cell proliferation and migration Collagen production Anti-inflammatory	[99]
AgNPs dressing	Spherical; 25.92 nm	Anti-inflammatory by inhibiting cytokine production ECM production	[100]
AgNPs dressing	Spherical; 50–90 nm	Anti-inflammatory by inhibiting the expression of pro-inflammatory cytokines Cell proliferation and migration	[101]
AgNPs ointment	86.38 nm	Antioxidant activity by significant reduction in secondary oxidation product (MDA) content and increased peroxidase activity Anti-inflammatory effects via downregulation of pro-inflammatory cytokine expression and upregulation of anti-inflammatory cytokine expression	[102]

**Table 5:** Applications of gold-based nanoparticles in wound healing.

Operation Method	Nanomaterial Properties	Biological Activity	References
AuNPs sponge	Spherical; 3.55 and 2.86 nm	Anti-inflammatory Skin tissue regeneration Cell proliferation	[122]
AuNPs ointment	1–3 nm, 3–5 nm, and 15–30 nm.	Cell proliferation Anti-inflammatory Antioxidant	[123]
AuNPs hydrocolloid membrane	30 nm	Antioxidant Tissue regeneration Stimulation of collagen synthesis through MMP-1 expression inhibition Stimulation of angiogenesis via differential regulation of related proteins	[124]
AuNPs ointment	Spherical; 15 nm.	Cell proliferation Cell migration Antioxidant Angiogenesis	[125]
AuNPs smear	Spherical; 20 nm	Anti-inflammatory Angiogenesis Stimulation of collagen synthesis	[126]
AuNPs gauze	Spherical; 13.2 nm	Anti-inflammatory Tissue regeneration Angiogenesis	[127]

**Table 6:** Applications of copper-based nanoparticles in wound healing.

Operation Method	Nanomaterial Properties	Biological Activity	References
CuNPs composite	Spherical; 50 nm	Antioxidant Anti-inflammatory Cell proliferation and migration Angiogenesis Collagen synthesis	[153]
CuNPs hydrogel	Spherical; 10 nm	Cell proliferation and migration Anti-inflammatory Collagen secretion Anti-inflammatory Angiogenesis	[154]
CuNPs hydrogel	Spherical; 88 nm	Anti-inflammatory Angiogenesis	[150]
CuNPs	Spherical; 40–80 nm	Cell proliferation and migration Collagen production Angiogenesis	[21]
CuNPs	100 nm	Angiogenesis	[155]

## Data Availability

The data presented in this study are available in the references listed below.
